# Affective Symptoms and Oropharyngeal Dysphagia in Head-and-Neck Cancer Patients: A Systematic Review

**DOI:** 10.1007/s00455-022-10484-8

**Published:** 2022-07-07

**Authors:** Iris Krebbers, Walmari Pilz, Sophie Vanbelle, Rob J. C. G. Verdonschot, Laura W. J. Baijens

**Affiliations:** 1 Department of Otorhinolaryngology, Head and Neck Surgery, Maastricht University Medical Center, P.O. Box 5800, 6202 AZ Maastricht, The Netherlands; 2 School for Oncology and Developmental Biology—GROW, Maastricht University Medical Center, Maastricht, The Netherlands; 3 School for Mental Health and Neuroscience—MHeNs, Maastricht University Medical Center, Maastricht, The Netherlands; 4 Department of Methodology and Statistics, Maastricht University, Maastricht, The Netherlands; 5 Care and Public Health Research Institute—CAPHRI, Maastricht University Medical Center, Maastricht, The Netherlands; 6 Emergency Department, Erasmus Medical Center, Rotterdam, The Netherlands

**Keywords:** Head-and-neck cancer, Affective symptoms, Anxiety, Depression, Deglutition disorders, Oropharyngeal dysphagia

## Abstract

Oropharyngeal dysphagia (OD) is a high impact morbidity in head-and-neck cancer (HNC) patients. A wide variety of instruments are developed to screen for affective symptoms and OD. The current paper aims to systematically review and appraise the literature to obtain insight into the prevalence, strength, and causal direction of the relationship between affective symptoms and OD in HNC patients. This review was conducted in accordance with the PRISMA statement. A systematic search of the literature was performed using PubMed, PsycINFO, Cochrane, and Embase. All available publications reporting on the relationship between affective conditions and swallowing function in HNC patients were included. Conference papers, tutorials, reviews, and studies with less than 5 patients were excluded. Fifteen studies met the inclusion criteria. The level of evidence and methodological quality were assessed using the ABC-rating scale and QualSyst critical appraisal tool. Eleven studies reported a positive relationship between affective symptoms and OD. The findings of this paper highlight the importance of affective symptom screening in dysphagic HNC patients as clinically relevant affective symptoms and OD seems to be prevalent and coincident in this population. Considering the impact of affective symptoms and OD on patients’ daily life, early detection and an integrated interdisciplinary approach are recommended. However, due to the heterogeneity of study designs, outcomes, and outcome measures, the generalization of study results is limited.

## Introduction

Swallowing is a complex neurocognitive process. It relies on accurate coordination of a variety of muscle and nerve groups aiming at efficient bolus preparation and safe and efficient bolus transfer from the oral cavity and pharynx to the esophagus [1]. Damage of upper aerodigestive tract tissue due to a head-and-neck malignancy or its oncological treatment may cause oropharyngeal dysphagia (OD) [2, 3]. The incidence of head-and-neck cancer (HNC) is rising, partly due to increasing numbers of human papilloma virus (HPV)-related oropharyngeal cancer resulting in a growing population of patients with a need for long-term healthcare [4]. OD is a high impact morbidity in head-and-neck cancer (HNC) patients with a reported prevalence of 45% [2, 5]. OD can be accompanied by severe complications such as aspiration pneumonia, sepsis, or malnutrition [3, 6]. Therefore, early screening, diagnosis, and treatment are essential to minimize the consequences of OD.

Besides affecting overall health, HNC may also affect mental health, social functioning, and employment [2]. This range of issues may have major effects on social and psychological well-being. The diagnosis and treatment of HNC itself may result in a significant burden on the patients’ psychological state because patients often find themselves in an ‘existential crisis situation’ [7]. Moreover, OD is often accompanied by anxiety, depression, reduced self-esteem, and social isolation, further amplifying the HNC-related suffering [6]. The recognition and treatment of the psychosocial burden in patients with HNC is important as distress may interfere with the ability to cope with the disease, its oncological treatment, and rehabilitation. The increasing incidence of HNC, combined with a high prevalence of psychological comorbidity in HNC patients [8], emphasizes the importance of an interdisciplinary approach including mental health care.

A wide variety of instruments are developed to screen for affective (anxiety and depression) symptoms and swallowing dysfunction. Screening tools are used for early identification of individuals at potentially high risk for a specific disorder. A screening tool such as the hospital anxiety and depression scale (HADS) is useful to identify clinically relevant anxiety and depressive symptoms [9]. For further identification of the nature and severity of a psychological disorder, a neuropsychological diagnostic workup is required. Regarding swallowing function, a screening tool such as the water swallow test (WST) is a quick and non-invasive method to identify patients at risk for unsafe swallowing [10]. After positive screening for OD, a clinical examination by a speech and language pathologist and/or instrumental swallowing assessment are recommended. In the literature, fiberoptic endoscopic evaluation of swallowing (FEES) and videofluoroscopic swallow study (VFSS) are considered the golden standard examinations to assess the swallowing function [11]. Besides clinician-reported outcome (CRO), self-evaluation is covered by patient-reported outcome (PRO) questionnaires. These questionnaires can roughly be divided into two different concepts: health-related quality of life (HRQoL) versus functional health status (FHS) questionnaires. FHS is often defined as one’s ability to perform daily activities required to meet basic needs, fulfill usual roles, and maintain their health and well-being [12]. HRQoL is a multi-dimensional concept that includes domains related to physical, mental, emotional, and social functioning [13]. HRQoL focuses on the impact of health status on quality of life [11, 14]. The concepts of HRQoL and FHS are often mixed, making it difficult to distinguish between tools that measure disease-related-QoL and functioning.

The aim of the present study is to systematically review and appraise the literature to obtain insight into the prevalence, strength, and causal direction of the relationship between OD and clinically relevant affective symptoms in HNC patients. A better understanding of this relationship will contribute to a better interdisciplinary approach to both problems (OD and affective symptoms) that can adversely affect each other during oncological treatment and rehabilitation.

## Methods

### Selection Process

The search strategy was developed using the PICO (Population, Intervention, Comparison, and Outcome) format as described in Table 1 [15]. To ensure an accurate and comprehensive capture of the study aims, a systematic literature search was carried out together with a university librarian, using four electronical databases (PubMed, Cochrane Library, Embase, and PsycINFO) on January 3, 2022. The databases were searched from January 1980 to December 2021.

**Table 1:** PICO

Population	Patients with head-and-neck cancer
Intervention	None
Comparison	None
Outcome	Having oropharyngeal dysphagia and affective symptoms

The methodology and reporting of this review were carried out according to the Preferred Reporting Items for Systematic Reviews and Meta-Analyses (PRISMA) statement [16]. Medical subject headings as well as free text words with truncation were used. Full search strategies specific to each database are described in Table 2. Two blinded independent reviewers included abstracts if the following criteria were met: (1) reporting on affective conditions (anxiety, depression, or emotional status), (2) reporting on swallowing function, (3) reporting on affective symptoms in relation to swallowing function, (4) in a population of patients with mucosal squamous cell carcinoma of the head and neck, (5) published in English, Dutch, German, Portuguese, Spanish, French, and (6) full-text retrievable. Conference papers, tutorials, reviews, duplicates, and studies with less than five patients were excluded. The same blinded independent reviewers screened full-text articles according to the same abstract inclusion criteria. The reference lists of the selected articles were hand screened for additional literature. The level of agreement between the two reviewers for eligibility after full-text screening was determined using percentage of agreement and Cohen’s kappa (κ). Discrepancies in article selection were resolved by consensus discussion.

**Table 2:** Syntax of the literature search

PubMed	(“Head and Neck Neoplasms”[Mesh] OR Head and neck Neoplasm* OR Upper Aerodigestive Tract Neoplasm* OR Cancer of the Head and Neck OR HNC OR Head and neck cancer) AND (dysphag* OR deglut* OR swallow* OR "Deglutition Disorders"[Mesh]) AND ((Psychiatr* OR depressi*) OR ((mood OR anxi* OR affective) AND (disorder* OR symptom*)) OR neuropsycho*):ti,ab
Embase	(Head and neck cancer or Head and neck Neoplasm* or Upper Aerodigestive Tract Neoplasm* or HNC or Cancer of the Head and Neck) and (dysphag* or deglut* or swallow*) and (Psychiatr* or depressi* or ((mood or anxi* or affective) and (disorder* or symptom*)) or neuropsycho*):ti,ab
PsycINFO	(Head and Neck Neoplasm* OR Cancer of the Head and Neck OR HNC OR Head and neck cancer* OR Upper Aerodigestive Tract Neoplasm) AND (dysphag* OR deglut* OR swallow*) AND (Psychiatr* OR depressi* OR ((mood OR anxi* OR affective) AND (disorder* OR symptom*)) OR neuropsycho*).ti,ab
Cochrane	(Head and neck cancer OR Head and neck Neoplasm* OR Upper Aerodigestive Tract Neoplasm* OR Cancer of the Head and Neck OR HNC) AND (dysphag* OR deglut* OR swallow*) AND (Psychiatr* OR depressi* OR ((mood OR anxi* OR affective) AND (disorder* OR symptom*)) OR neuropsycho*):ti,ab

### Level of Evidence and Critical Appraisal

The level of evidence of all included studies was assessed using the ABC-rating scale [17]. In this scale, level A refers to high‐quality randomized controlled trials and meta-analysis; level B refers to nonrandomized clinical trials, nonquantitative systematic reviews, and clinical cohort studies, and level C refers to consensus viewpoints and expert opinions.

Subsequently, the reviewers independently appraised the included articles for methodological quality according to the QualSyst critical appraisal tool [18]. The QualSyst tool is developed for the quality assessment of both qualitative and quantitative studies using any study design. The QualSyst tool for standard quality assessment of quantitative studies is a validated checklist that is made up of 14 criteria to be assessed including research questions and objectives, study design, subject and comparison group selection and characteristics, interventional allocation, definitions of outcomes, sample size, analytic methods, confounding, reported results, and conclusions. The scores of each item range from 0 to 2 with a maximum total QualSyst score being 28. A summary score can be obtained by dividing the total score by the total possible score [.e., 28 − (number of not applicable items × 2)]. According to the QualSyst tool, the methodological quality of the articles can be classified as limited < 0.50, adequate 0.50–0.70, good 0.70–0.80, and strong > 0.80 [19]. The level of agreement between the two reviewers for the ABC-rating scale and the QualSyst critical appraisal tool was obtained using percentage of agreement.

### Data Extraction

Both independent reviewers extracted relevant data into summary tables (Tables 3 and 4). Extracted data included sample size, study population (etiology, age, and sex), method of OD and affective symptom assessment, timing of assessment, and study results according to the authors. Descriptive summaries were generated, including the exploration of relationships in the data. Finally, critical reflection of this review process was described in the discussion section of this paper. Radiographic procedure that provides a﻿ dynamic view of oral, pharyngeal, and upper esophageal function during swallowing.

## Results

**Fig. 1:**
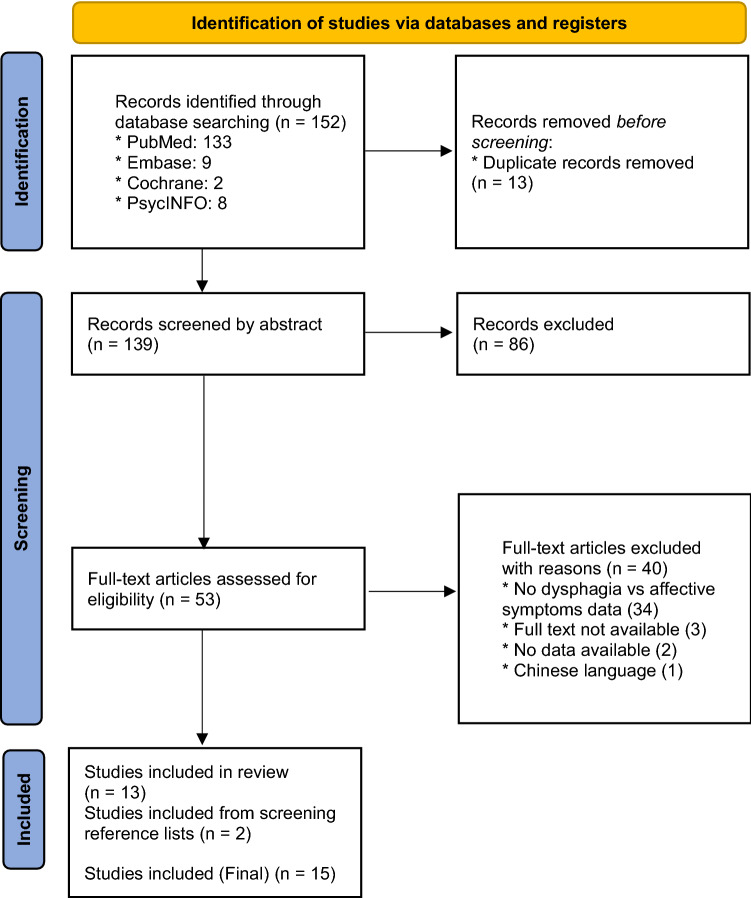
PRISMA flowchart of the literature review process

The systematic searches across all databases yielded a total of 139 abstracts after duplicate references were removed. Of these, 86 abstracts were removed based on the exclusion criteria. The full-text of the remaining 53 abstracts was reviewed. Two articles were identified after hand searching the reference lists of the included studies. Finally, 15 articles were included in this systematic review. The PRISMA flow diagram illustrates the search process (Fig. 1).

The two reviewers had 78% agreement (κ = 0.55) on the selection based on title and abstract screening. Articles that were selected by just one of the reviewers were subsequently screened using the full-text. At the full-text review stage, the two reviewers had 84% agreement (κ = 0.63) on their ratings. The two reviewers had 100% agreement on the ABC-rating scale and 93.3% on the QualSyst ratings. Disagreements were discussed and resolved in consensus.

### Methodological Quality of Included Studies

All studies met the criteria of level B according to the ABC-rating scale (eight cross-sectional studies, six clinical cohort studies, and one case–control study) [17]. The methodological quality of the studies based on the QualSyst ratings ranged from adequate (0.68) to strong (0.96). Seven articles were ranked as strong [20–26], four as good [27–30], and four as adequate [25, 31–33]. The level of evidence and methodological quality of the 15 articles are presented in Table 4.

### Swallowing Function and Affective Symptoms

Swallowing function was investigated by a range of CRO tools such as OD screening tools (WST) and CRO tools such as VFSS and FEES [10, 34, 35]. Visuoperceptual ordinal variables on swallowing safety and efficiency, the dysphagia outcomes and severity scale (DOSS), the swallowing performance scale (SPS), and the penetration–aspiration scale (PAS) were the outcome measures used for VFSS and FEES [36–38]. Patients’ perception of the swallowing function was evaluated using the following PRO HRQoL questionnaires: the global, functional, and physical subscales of the MD Anderson dysphagia inventory (MDADI), the swallowing domain of the University of Washington quality of life (UW-QOL), the swallowing domain of the Dische morbidity recording scheme, and the swallowing domain of the European organization for research and treatment for cancer quality of life questionnaire head and neck module (EORTC QLQ-H&N35) [39–42].

Affective symptoms were measured using CRO or PRO questionnaires. The majority of the questionnaires was specifically developed to measure depression or anxiety symptoms such as the hospital anxiety and depression scale (HADS), hospital Beck depression inventory fast screen (BDI-FS), depression anxiety stress score (DASS), Montgomery Asberg depression rating scale (MADRS), and the Zung self-rating depression scale (SDS) [9, 43–46]. Questionnaires with a domain reporting on patients’ emotional status, such as the mood and anxiety domains of the UW-QOL (version 4) and the emotional domain of the MDADI, were also included in this systematic review [47]. The most frequently used PRO measure was the HADS followed by the MDADI and the EORTC QLQ-H&N35 questionnaires.

FHS was measured using the performance status scale for head-&-neck cancer patients (PSS-HN), functional assessment of cancer therapy-general, functional assessment of cancer therapy-head and neck questionnaires (FACT-G and FACT-H&N) [48–50]. An overview of the characteristics and validation of the tools used to screen or measure swallowing function and affective symptoms in the included articles is presented in Table 3.

**Table 3:** Overview of the assessment tools

Reference	Assessment tool	Type	Short description	Validation
	OD	PRO		
[42]	European organization for research and treatment of cancer quality of life questionnaire head and neck module (EORTC QLQ-H&N35, supplementary module to EORTC QLQ-C30)	PRO HRQoL	A 35-item questionnaire to assess HRQoL specifically in HNC patients. It has seven multi-item scales (pain, swallowing, senses, speech, social eating and social contact, and sexuality) and eleven single-item scales (teeth, mouth opening, dry mouth, sticky saliva, coughing, feeling ill, pain killers, nutritional supplements, feeding tube, weight loss, and weight gain)	Validated for HNC
[39]	MD Anderson dysphagia inventory (MDADI)	PRO HRQoL	A 20-item questionnaire to evaluate the emotional, physical, and functional impact of OD in HNC patients The questionnaire is divided in four subscales (global, functional, physical, and emotional). Each item is rated on a 5-point scale with higher scores indicating better function	Validated for HNC
[40]	University of Washington quality of life (UW-QOL) version 3	PRO HRQoL	A HRQoL measurement in HNC patients. The questionnaire measures ten domains: pain, appearance, activity, recreation, swallowing, chewing, speech, shoulder function, taste, saliva, and global overall assessment of QOL. A composite score is calculated ranging from 0 (worst possible response) to 100 (best possible response)	Validated for HNC
[41]	Dische morbidity recording scheme	PRO	Scoring scale of radiation-induced late-effect changes in the oral and/or pharyngeal mucosal and/or salivary glands. Dysphagia is one of the three symptom-subscales and is classified as a 4-point scale ranging from normal function (0) to severe difficulty with swallowing fluids (4) based on a visual analog scale from 0 (no impairment) to 10 (maximum impairment)	Not validated

	OD	CRO		
[36, 37]	Dysphagia outcomes and severity scale (DOSS)/swallowing performance scale (SPS) during	CRO based on functional assessment during instrumental swallowing evaluation	7-point scale to rate the functional severity of OD based on VFSS and make recommendations for diet level, independence level, and type of nutrition. The scale ranges from one (DOSS: severe dysphagia; SPS: normal swallowing) to seven (DOSS: normal swallowing; SPS: severe dysphagia)	Not validated
[34]	﻿Videofluoroscopic swallow study (VFSS)			
[38, 56]	Visuoperceptual ordinal variables on swallowing safety and efficiency/penetration-aspiration scale (PAS) during	CRO based on functional assessment during instrumental swallowing evaluation	Visuoperceptual ordinal variables as piecemeal deglutition, post-swallow vallecular pooling, post-swallow pyriform sinus pooling, penetration, and aspiration	Not validated
[35]	Fiberoptic endoscopic evaluation of swallowing (FEES)		Procedure in which a transnasal fiberoptic endoscope is inserted into the pharynx to evaluate the structures and bolus transfer during swallowing	
[10]	Water swallow test (WST)	CRO based on functional assessment during clinical swallowing screening	Swallowing screening test where a patient is offered 30 mL of water. The time and number of swallows required to drink the entire 30 mL is recorded as well as presence of cough or choking	Not validated
[26]			The scale used in the study from Zhang et al. ranges from I (normal swallowing) to V (being unable to swallow)	

	﻿Affective symptoms	PRO		
[45]	Beck depression inventory fast screen (BDI-FS)	PRO QoL	A 7-item questionnaire to assess cognitive and affective aspects of depression ranging in intensity Each item is rated on a 4-point scale and higher scores are indicative of more severe symptoms	Validated for other population but not for HNC
[43]	Depression anxiety stress score (DASS)	PRO QoL	A 42-item questionnaire to screen for anxiety, depression, and stress. Each item is rated on a 4-point scale and higher scores are indicative of more symptoms	Validated for other population but not for HNC
[9]	Hospital anxiety and depression scale (HADS)	PRO HRQoL	A 14-item questionnaire to screen for clinically relevant symptoms of anxiety and depression. Each item is rated on a 4-point scale and higher scores represent a higher risk of affective symptoms	Validated for other population but not for HNC
[46]	Zung self-rating depression scale (SDS)	PRO QoL	A 20-item questionnaire to assess affective, psychological, and somatic symptoms associated with depression. Each item is scored on a 4-point scale and higher scores are indicative of more severe depression	Validated for other population but not for HNC
[47]	University of Washington quality of life (UW-QOL) version 4	PRO HRQoL	A HRQoL measurement in HNC patients. In version 4, the mood and anxiety domains are added A composite score is calculated ranging from 0 (worst possible response) to 100 (best possible response)	Validated for HNC

	﻿Affective symptoms	CRO		
[44]	Montgomery Asberg depression rating scale (MADRS)	CRO QoL	A 10-item questionnaire to measure the severity of depressive symptoms based on a non-standardized interview investigating areas including emotional, cognitive, and physical symptoms. Each item is rated on a 7-point scale and higher scores are indicative of more severe symptoms of depression	Validated for other population but not for HNC

	﻿FHS			
[49]	Functional assessment of cancer therapy-general (FACT-G)	PRO FHS	A 27-item questionnaire designed to measure four domains in cancer patients: Physical, social, emotional, and functional well-being. Each item is scored on a 5-point scale and higher scores indicate a better functional health state	Validated for other population but not for HNC
[50]	Functional assessment of cancer therapy-head and neck (FACT-H&N)	PRO FHS	A 39-item questionnaire including the FACT-G questionnaire and 12 general items related to HNC (swallowing, voice, disfigurement, tobacco, alcohol, communication) Each item is scored on a 5-point scale and higher scores indicate a better functional health state	Validated for HNC
[48]	Performance status scale for head and neck cancer patients (PSS-HN)	CRO FHS	3-domain questionnaire evaluating normalcy of diet, eating in public, and speech in HNC patients. Each item is rated on a scale from 0 to 100 and higher scores indicate better performance	Validated for HNC

*CRO* clinician-reported outcome, *FHS* functional health status, *HNC* head and neck cancer, *HRQoL* health-related quality of life, *OD* oropharyngeal dysphagia, *PRO* patient-reported outcome, *QoL* quality of life

Table 4 summarizes the data retrieved from the included articles regarding sample size, oncological treatment modalities, measurements tools, outcome measures, and the reported relationship between swallowing function and affective symptoms or emotional status. The sample size of the included studies ranged from 9 to 110. The mean age of the patients in the included studies ranged from 27 to 83 and most of the included patients were male. Eleven studies included a heterogeneous mix of head and neck tumor locations, one study only included patients with oral cavity cancer, another study only included patients with oropharyngeal cancer, and two studies only included patients who had undergone a total laryngectomy. Surgery was the most frequently applied oncological treatment, and in some studies, this treatment modality was followed by adjuvant (chemo)radiotherapy. In the majority of the studies, swallowing function and affective symptoms were evaluated after the oncological treatment was ended (mean duration of time interval: 38 months (range 1–63 months).

**﻿Table 4:** Overview of the included studies

﻿References	Level of evidence and QualSyst score	N	Measurement tools used to screen or assess affective symptoms and OD	Reported results in the included studies
		N patients with OD population Moment of evaluation		
Airoldi et al. [27]	B (Cross-sectional study)	N = 36 N(OD) = 23	Affective symptoms: HADS MADRS	A significant correlation between OD severity (Dische) and symptoms of anxiety and depression (HADS) was observed (depression *r* = 0.389, *p* = 0.019; anxiety *r* = 0.387, *p* = 0.02). Moreover, patients with severe OD (Dische grade 3–4) showed significantly higher symptom levels of anxiety and depression compared to patients without or mild OD (Dische grade 0–1) No association was found between Dische and MADRS
	0.73 (Good)	Oral cavity cancer survivors (surgery followed by adjuvant radiotherapy) Median 63 months post-treatment	OD: Dische morbidity recording scheme	
Bozec et al. [28]	B (Prospective cohort study)	N = 58 N(OD) = 28	Affective symptoms: HADS	Psychological distress (HADS scores) was an independent predictor of swallowing impairment (DOSS scores). Psychological distress (higher HADS-depression and total scores) was significantly associated with a poorer swallowing function (lower DOSS score) (*p* = 0.01 and *p* = 0.04, respectively)
	0.77 (Good)	Oropharynx cancer survivors (surgery with or without adjuvant (chemo)radiotherapy) Pre- and post-treatment (mean 54 months post-treatment)	OD: DOSS (based on VFSS)	
Campbell et al. [29]	B (Cross-sectional study)	N = 62 N(OD) = 27	OD: PAS (based on VFSS)	Patients without aspiration during VFSS reported better emotional well-being (higher FACT-G emotional scores) compared to patients who aspirated; however, this association was not significant (*p* = 0.17). Aspiration during VFSS was associated with a worsened global score of additional head and neck concerns on the FACT-H&N scale (decreased health state) (*p* ≤ 0.001)
	0.79 (Good)	HNC survivors (surgery and/or radiotherapy) At least 5 years post-treatment	FHS: FACT-G FACT-H&N PSS-HN	
Chan et al. [20]	B (Prospective cohort study)	N = 77 N(OD) = not reported	Affective symptoms: BDI-FS	Depression symptom scores (BDI-FS) were significantly associated with the MDADI-functional (*β* = 17.31; *p* = 0.009) and physical (*β* = 14.99; *p* = 0.032) subscales
	0.96 (Strong)	HNC survivors Prior to treatment	OD: MDADI	
Cnossen et al. [25]	B (Prospective cohort study)	N = 67 N(OD) = 27	Affective symptoms: HADS	Patient-reported swallowing problems (EORTC QLQ-H&N35) were significantly related to emotional distress (HADS) at the two time points measured in the study: diagnosis (*r* = 0.52, *p* = 0.00) and at the first follow-up visit (*r* = 0.46, *p* = 0.00)
	0.68 (Adequate)	HNC survivors (surgery with or without adjuvant (chemo)radiotherapy) At time of HNC diagnosis (baseline) and median 1-month post-treatment	OD: EORTC QLQH&N35	
Florie et al. [21]	B (Cross-sectional study)	N = 63 N(OD) = 63	Affective symptoms: MDADI-emotional subscale	Statistically significant mean differences of the MDADI- physical subscale between the ordinal scale levels of the FEES variable piecemeal deglutition (*p* = 0.043) were found and of the MDADI-general and functional subscales between the ordinal scale levels of post-swallow vallecular pooling (*p* = 0.020 and *p* = 0.018, respectively) for thick liquid swallows. These results indicate that a higher score on the ordinal FEES outcome scale (worse swallowing functioning) is accompanied by a lower score on the MDADI subscales (lower swallow-specific QOL). All other comparisons showed no statistically significant results
	0.96 (Strong)	HNC survivors (surgery, (chemo)radiotherapy, or combinations) Post-treatment	OD: MDADI FEES	
Hartl et al. [31]	B (Cross-sectional study)	N = 9 N(OD) = not reported	Affective symptoms: HADS	The HADS-depression was significantly correlated with the EORTC QLQ-H&N35-swallowing domain (*p* = 0.023). A trend toward a correlation between the HADS-depression and the EORTC QLQ-H&N35-aspiration domain was observed. However, this correlation was not statistically significant (*p* = 0.06)
	0.68 (Adequate)	Tongue(base) cancer survivors (surgery followed by adjuvant(chemo)radiotherapy) Median 43 months post-treatment	OD: EORTC QLQ-H&N35-swallowing and aspiration domains	
Kemps et al. [22]	B (Cross-sectional study)	N = 35 N(OD) = 35	Affective symptoms: HADS	Clinically relevant anxiety symptom scores on the HADS were significantly associated with the functional and physical domains of the MDADI (*p* = 0.006; *p* = 0.001; respectively). The same applies for clinically relevant depression symptom scores (*p* = 0.006; *p* < 0.001; respectively)
	0.86 (Strong)	HNC survivors (total laryngectomy with or without adjuvant (chemo)radiotherapy) Mean 85 months post-treatment	OD: MDADI-functional and physical domains	
Krebbers et al. [23]	B (Cross-sectional study)	N = 84 N(OD) = 84	Affective symptoms: HADS	There was a statistically significant association between aspiration identified during FEES and HADS-anxiety, HADS-depression, and HADS-total scores (*p* = 0.05, *p* = 0.04, *p* = 0.04). Patients presenting with aspiration scored on average 2.0, 2.2, and 4.2 points lower on the HADS-anxiety, HADS-depression, and HADS-total scale compared to dysphagic patients who did not aspirate, representing lower symptom scores for anxiety and depression
	0.96 (Strong)	HNC survivors (surgery, (chemo)radiotherapy, or combinations) Median 42 months post-treatment	OD: PAS (based on FEES)	
Lin et al. [30]	B (Prospective cohort study)	N = 46 N(OD) = not reported	Affective symptoms: BDI-FS	Compared to nondepressed patients, depressed patients (BDI-FS) reported significantly lower scores on the UW-QOL swallowing domain (*p* = 0.007) and on the MDADI-functional and physical domains (*p* = 0.001; *p* = 0.001, respectively), representing more severe swallowing complaints Multivariate logistic regression analysis of the UW-QOL and MDADI scores demonstrated an association between depression (BDI-FS) and UW-swallowing (B = − 23.9, *p* = 0.035) after controlling for sex, age, comorbidity, marital status, tumor stage, treatment, MDADI, VHI, and UW-QOL
	0.73 (Good)	HNC survivors (surgery, (chemo)radiotherapy, or combinations) 12 months post-treatment	OD: UW-QOL-swallowing domain MDADI-functional and physical domains	
Maclean et al. [24]	B (Case–control study)	N = 110 N(OD) = 79	Affective symptoms: DASS UW-QOL mood and anxiety domains	Laryngectomees who reported swallowing impairment on the questionnaire presented significantly higher levels of depression (*z* = − 2.58, *p* = 0.010), anxiety (*z* = − 2.94, *p* = 0.003), and stress (*z* = − 2.139, *p* = 0.032) on DASS, compared to laryngectomees who reported the absence of swallowing impairment. Laryngectomees who reported swallowing impairment had significantly worse mean scores on mood (*z* = − 3.39, *p* = 0.001), and anxiety (*z* = − 2.75, *p* = 0.006) domains of the UW-QOL, compared to laryngectomees who report absence of swallowing impairment
	0.82 (Strong)	HNC survivors (total laryngectomy with or without adjuvant (chemo)radiotherapy) Post-treatment	OD: Self-designed demographic questionnaire including items on swallowing: –Any difficulty in swallowing? (yes/no) –Changes to their diet texture? (yes/no) –Patients had to list any foods avoided and state why	
Nguyen et al. [32]	B (Retrospective cohort study)	N = 104 N(OD) = 73	Affective symptoms: ﻿HADS	Patients without or mild OD identified during VFSS (G1-2) presented lower levels of anxiety (*p* = 0.005) and depression (*p* = 0.0001) symptoms (HADS) compared to patients with moderate or severe OD (G3–4)
	0.68 (Adequate)	HNC survivors (surgery, (chemo)radiotherapy, or combinations) G1: No OD N = 31 G2: Mild OD N = 24 G3: Moderate OD N = 25 G4: Severe OD N = 24 ﻿Median 24 months post-treatment	OD: ﻿SPS (based on VFSS)	
Verdonck- de﻿ Leeuw et al. [51]	B (Cross-sectional study)	N = 45 N(OD) = 7	Affective symptoms: HADS OD: Presence/absence of a ﻿feeding tube	Patients who were feeding tube-dependent had significantly (*p* < 0.05) higher HADS-total scores (mean 14.9; SD 10.7) compared to patients with oral feeding (mean 8.8; SD 6.0). No specific information on the association between OD and the HADS subscales anxiety a﻿nd depression was provided
	0.82 (Strong)	HNC survivors (surgery, (chemo)radiotherapy, or combinations) Mean ﻿29 months post-treatment		
Zhang et al. [26]	B (Prospective cohort study)	N = 58 N(OD) = 58	Affective symptoms: ﻿SDS	Lower levels at the WST (I normal swallowing and II doubtful OD) were associated with lower SDS scores (lower symptom levels of depression). Before swallowing training, WST levels and SDS scores were significantly higher (the presence of OD and higher symptom levels of depression) than those measured after swallowing training and they tended to co-occur in the same direction
	0.86 (Strong)	Tongue(base) cancer survivors (surgery) Before and after ten days of postoperative swallowing training	OD: WST	
﻿Zwahlen et al. [33]	B (Cross-sectional study)	N = 31 N(OD) = not reported	Affective symptoms: ﻿HADS	Patient-reported swallowing problems (EORTC QLQ-H&N35-swallowing domain) were correlated to higher symptom levels of anxiety and depression (*p* = 0.24; *p* = 0.30); however, this relationship was not clinically significant
	0.68 (Adequate)	HNC survivors (surgery with or without adjuvant (chemo)radiotherapy) Mean 44 months since cancer diagnosis	OD: EORTC QLQH&N35-swallowing domain	

*BDI-FS* Beck depression inventory fast screen, *DASS* Depression anxiety stress score, *DOSS* Dysphagia outcomes and severity scale, *EORTC QLQH&N*35 European organization for research and treatment of cancer quality of life questionnaire head and neck module, *FACT-G* Functional assessment of cancer therapy-general, *FACT-H&N* Functional assessment of cancer therapy-head and neck, *FEES* Fiberoptic endoscopic evaluation of swallowing, *FHS* functional health status, *HADS* Hospital anxiety and depression scale, *HNC* head and neck cancer, *MADRS* Montgomery Asberg depression rating scale, *MDADI* MD Anderson dysphagia inventory, *OD* oropharyngeal dysphagia, *PAS* penetration-aspiration scale, *PSS-HN* Performance status scale for head-and-neck cancer patients, *QoL* quality of life, *SDS* Zung self-rating depression scale, *SPS* Swallowing Performance Scale, *UW-QOL* University of Washington quality of life, *VFSS* Videofluoroscopic swallow study, *WST* Water swallow test

In the included studies, the prevalence of OD ranged from 16 to 100%. The reported prevalence for clinically relevant affective symptoms ranged from 12 to 54%. A positive relationship between OD and affective symptoms or emotional status was described in most of the studies [20, 22, 24–28, 30–32, 51]. Four studies found a non-significant or negative relationship between swallowing function and affective symptoms or emotional status [21, 23, 29, 33]

## Discussion

The initial purpose of the present study was to systematically review and appraise the literature on the prevalence of affective symptoms and to identify the strength and direction of the relationship between OD and clinically relevant affective symptoms in HNC patients. A better understanding of this relationship will contribute to a better interdisciplinary approach to both problems (OD and affective symptoms) that can adversely affect each other during oncological treatment and rehabilitation. In general, OD and affective symptoms were related to each other as described below. However, the results of this systematic review have not been able to adequately answer the question on the strength and direction of this relationship.

In total, 15 articles were included. The methodological quality of the included studies ranged from adequate (0.86) to strong (0.96). Due to the heterogeneity of study designs, terminology, outcomes, and outcome measures used, a meta-analysis could not be conducted.

A positive relationship between OD and affective symptoms or emotional status was described in the majority of the studies [20, 22, 24–28, 30–32, 51]. Nguyen et al. reported that HNC patients experience anxiety and depression related to their OD severity which can be explained by the functional impairment and disfigurement resulting from HNC and its treatment [30]. Eating and drinking is an important part of social interaction, but dysphagic HNC patients often experience eating difficulties in public and home environment [23]. In some cases, this may lead to exclusion of invitations or to patients declining to eat out. Therefore, patients may become socially isolated leading to symptoms of anxiety and depression. Maclean et al. and Zhang et al. described that an improvement in swallowing function may reduce the severity of affective symptoms in HNC patients [28, 30]. On the other hand, affective symptoms can cause physical complaints such as dry mouth which may enhance swallowing impairment [45]. Furthermore, affective symptoms can negatively affect motivation and consequently compliance during cancer rehabilitation, resulting in a poor functional outcome [50]. Bozec et al. described that depressive symptom scores are independent predictors of poorer swallowing function in HNC patients, highlighting the fact that swallowing function is highly dependent on psychological, emotional, and social conditions in addition to tumor or treatment characteristics [26]. In addition, the patient’s psychological baseline should be taken into account as affective symptoms may already be present prior to the HNC diagnosis [26].

Although a positive relationship between OD and affective symptoms has been described in the majority of the included studies, other studies found a non-significant or negative relationship between swallowing function versus anxiety, depression, or emotional status [21, 23, 29, 33]. The reasons for these divergent findings can be multiple. For example, the timing of assessment of OD and affective symptoms varies widely between these studies. The moment of measurement plays an important role in the outcome of swallowing-, physical-, and emotional functioning. The longitudinal study of Hammerlid et al. reported that patients may develop coping skills or undergo changes in the experience of the disease and in their expectations of health over time resulting in improved symptom scores [51]. These findings justify the recommendation to systematically screen for affective symptoms and swallowing disorders at baseline (before oncological treatment) and during the oncological follow-up.

A variety of tools measuring swallowing function and affective symptoms were used in the included studies. These different tools vary in use and purpose (screening versus diagnostic) and the interpretation and clinical relevance of the outcome measures should be taken into account. Multiple PRO HRQoL questionnaires were used to measure patients’ perception of the swallowing function based on the multi-dimensional concept of HRQoL including domains related to physical, mental, emotional, and social functioning. However, only one study included PRO FHS questionnaires to determine the impact of OD on the ability to perform daily activities [29]. In a cross-sectional study, Campbell et al. aimed to determine associations between instrumental assessment (VFSS), PRO HRQoL measurement (UW-QOL), and FHS measurements (FACT-G, FACT-H&N and PSS-HN) in HNC survivors, 5 years post-treatment [29]. Patients presenting aspiration of the bolus into the airway scored significantly lower on the UW-QOL swallowing domain, FACT-H&N additional concerns, and PSS-HN ‘normalcy of diet’ domain, compared to non-aspirators. Aspiration was not associated with PSS-HN ‘willingness to eat in public’ domain nor with any of the FACT-G well-being scales. So, despite unsafe swallowing (VFSS) and poor swallowing-related HRQoL (UW-QOL), aspiration of the bolus into the airway does not seem to impact everyday activities and fulfilling usual roles.

It is not uncommon to find a discrepancy between the results of PRO and CRO measures [52]. Airoldi et al. reported on a discrepancy between a self-reported prevalence of depressive symptoms of 30% measured using the HADS versus a clinician-reported prevalence of 44.4% measured by the MADRS in a population of patients following treatment for oral cancer [27]. The authors concluded that this discrepancy might be related to an inadequate self-awareness of HNC patients concerning illness-related psychological distress. Furthermore, according to the authors, a higher prevalence of having a vulnerable socioeconomic status, addictive behavior, and anosognosia can play a role in limited self-awareness in this patient population [27]. In addition, Florie et al. reported on the very few statistically significant mean differences of MDADI subscale scores between the ordinal scale levels of several FEES variables in a heterogenous population of HNC patients following cancer treatment. This study also described a weak relationship between the severity of OD and PRO OD-specific HRQoL. The authors concluded that adaptive changes in swallowing function, radiation neuropathy with a decreased oropharyngeal sensibility, and the patients’ noncomplaining nature and lack of initiative may affect the perception (underestimation) of their swallowing difficulties. That perception or underestimation, in turn, may determine their score on the MDADI questionnaire [21]. The MDADI was developed to measure the influence of OD on the patients’ HRQoL. Nevertheless, it still remains unclear if the MDADI can be used as an indicator for the severity of OD [21]. Although these PRO and CRO measures do not correlate well, it remains important to realize the existence of these different dimensions of OD as well as their application and relevance in both scientific research and daily clinical practice.

Non-validated measurement tools were used in eleven of the included studies. The use of high-quality measurement tools based on robust psychometric properties such as validity and reliability is strongly recommended and essential to accurately estimate the prevalence of affective symptoms and OD [53].

Although no meta-analytic conclusions can be drawn from the included articles, OD and affective symptoms often appear to be coincidental in HNC patients. The included studies reported a prevalence of OD ranging from 16 to 100% and a prevalence of clinically relevant affective symptoms ranging from 12 to 54%. This wide variation in the prevalence of OD and clinically relevant affective symptoms in the different studies may be due to several reasons: different study designs with various HNC patient samples (variation in age, tumor location, tumor stage, oncological treatment, etc.), the use of different and/or non-validated measuring instruments for OD and affective symptoms in HNC patients, different timing of measurement, etc.

Seven studies reported the effects of tumor location, tumor stage, and/or oncological treatment modality on swallowing, and affective symptoms [23, 26, 28–32, 51]. Depending on the tumor location, swallowing function may be affected in different ways. For instance, in the literature, a higher incidence of aspiration before the start of the oncological treatment is reported in patients with laryngeal and hypopharyngeal cancer [54]. However, the included studies of the present review did not report on the relationship between tumor location and OD severity nor on the relationship between tumor location and clinically relevant affective symptoms. A significant relationship between tumor stage versus OD and depressive symptoms was reported [26, 29, 30]. An advanced tumor stage can cause more severe OD and functional impairment due to a greater extent of damage to essential structures of the upper aerodigestive tract. However, this relationship was established after oncological treatment, so most likely the type and number of oncological treatment modalities play a role in this relationship. Advanced primary site disease often necessitates aggressive multimodality treatment, which puts patients at greater risk of long-term disability as a result of surgical and adjuvant (chemo)radiotherapy induced functional loss [30]. (Chemo)radiotherapy may induce mucositis, stomatitis, hyposalivation, trismus, soft tissue necrosis, fibrosis, and osteoradionecrosis of the mandible [27]. The effect of surgery on swallowing function and affective symptoms should also be considered as OD, and affective symptoms seem to be related to the location and extent of the resection. A significant association between the extent of tongue(base) resection versus OD and depressive symptoms was reported [26, 31]. However, six studies did not find any effect of tumor location, tumor stage, and type of oncological treatment modality on the prevalence of affective symptoms and OD showing that this relationship is not yet well understood [23, 25, 28, 32, 33, 51].

All included studies applied patient-reported questionnaires on OD and affective symptoms. However, only few studies screened or assessed the level of cognition of the included patients [21–23]. When using PRO tools, it is necessary to screen or estimate the level of cognition prior to completing a self-report questionnaire to guarantee that patients are able to understand and answer the questions accordingly. Cognitive impairment may complicate recall-based assessment with questionnaires resulting in recall bias. Besides cognition, alcohol consumption should also be reported when evaluating affective symptoms. Prolonged alcohol use is often seen in HNC patients and known to cause structural changes in the brain as well as cognitive deficits [55]. Four articles reported on a significant association between alcohol abuse and long-term psychological distress, resulting in a vicious circle in which these phenomena reinforce each other [27, 28, 30, 33]. This highlights the importance to support HNC patients with an active addiction. Finally, although the use of psychotropic drugs is likely to influence the severity of affective symptoms, only two studies reported on the use of psychotropic drugs [22, 23].

## Limitations and Risk of Bias

This systematic review has some limitations. The systematic search generated a low number of articles on affective symptoms and OD in HNC patients. Reasons for this low number may be related to the inconsistent terminology used in this research topic. The lack of randomized controlled trials and pre- and post-treatment data studies may limit the strength of the findings. All studies had methodological limitations (e.g., lack of details provided regarding selection criteria; small sample sizes; limited information on methods, incomplete information about measurement tools and procedures; no information about test result interpretation). The heterogeneity of the included studies is likely to have contributed to the overall variation in reported frequencies of OD and affective symptoms and precluded eligibility to pool data across studies.

## Conclusion

This study shows that screening for affective symptoms in dysphagic HNC patients should be considered as affective symptoms and OD seems to be prevalent and coincident in this population. The strength and direction of the relationship between affective symptoms and OD still remain unclear. Considering the impact of affective symptoms and OD on patients’ daily life, early detection and an integrated interdisciplinary approach are recommended. Future studies should use validated measurement tools, bigger sample sizes, and study designs that lead to high-quality evidence.
